# Parathyroid cyst associated with laboratory findings suggestive of normocalcemic primary hyperparathyroidism: a case report

**DOI:** 10.25122/jml-2026-0053

**Published:** 2026-09

**Authors:** Moaaz Amir, Salwa Sheikh

**Affiliations:** 1 College of Medicine, Imam Abdulrahman Bin Faisal University, Dammam, Saudi Arabia; 2 Department of Pathology, Johns Hopkins Aramco Healthcare, Dhahran, Saudi Arabia

**Keywords:** parathyroid diseases, cysts, hyperparathyroidism, primary, parathyroid hormone, osteoporosis, postmenopausal, parathyroidectomy

## Abstract

Parathyroid cysts are uncommon lesions and can be challenging to diagnose preoperatively. We report a 62-year-old postmenopausal woman assessed for osteoporosis following a low-impact ankle fracture, who was found to have persistently elevated intact parathyroid hormone, peaking at 101 pg/mL, with repeatedly normal serum calcium (10.1 mg/dL preoperatively). Neck ultrasound and technetium-99m sestamibi scintigraphy localized a solid-appearing lesion inferior to the left thyroid lobe, initially suspected to be a parathyroid adenoma. Left inferior parathyroidectomy was performed. The excised specimen measured 1.6 × 1.0 × 0.5 cm and contained a 1.3 cm cyst filled with clear fluid; microscopic examination showed a cyst lined by flattened to cuboidal parathyroid cells without malignant features, establishing the diagnosis of a parathyroid cyst. Parathyroid hormone fell to 50.1 pg/mL postoperatively (reference range, 10–65 pg/mL) and serum calcium remained normal. This case illustrates the difficulty of distinguishing parathyroid cysts from adenomas preoperatively and emphasizes the importance of considering parathyroid cysts in patients with hyperparathyroidism despite normal serum calcium.

## INTRODUCTION

Parathyroid cysts are rare lesions, accounting for fewer than 1% of all parathyroid pathology [1]. They typically lack obvious clinical symptoms and are commonly misdiagnosed as thyroid nodules or parathyroid adenomas on radiologic evaluation [1,2]. Although most parathyroid cysts are non-functional, functional cysts causing hyperparathyroidism have been described [1]. The diagnosis is often confirmed postoperatively, as preoperative biochemical and radiologic findings may overlap with those of parathyroid adenomas [2,3]. Normocalcemic primary hyperparathyroidism is an increasingly recognized entity defined by elevated parathyroid hormone despite normal serum calcium, after exclusion of secondary causes [4,5]. The concurrence of normocalcemic primary hyperparathyroidism and a parathyroid cyst is rarely encountered and may pose diagnostic challenges [1,4]. We report a case of a parathyroid cyst with features suggestive of normocalcemic primary hyperparathyroidism and associated skeletal complications, initially presumed on imaging to be a parathyroid adenoma.

## CASE PRESENTATION

A 62-year-old postmenopausal woman was evaluated in the endocrinology clinic for osteoporosis following a low-impact fracture of the distal left fibula and lateral malleolus—a fragility fracture that establishes the clinical diagnosis of osteoporosis irrespective of densitometry. Her medical history included type 2 diabetes mellitus, hypertension, and hyperlipidemia. She had no history of nephrolithiasis, no family history of hypercalcemia or endocrine disorders, and was a non-smoker.

Dual-energy X-ray absorptiometry performed over several years demonstrated a gradual reduction in bone mineral density (BMD). Left femoral neck BMD was 0.775 g/cm^2^ (T-score, −1.9) and lumbar spine BMD was 0.926 g/cm^2^ (T-score, −2.1). The 10-year fracture probability estimated with the Fracture Risk Assessment Tool was 16.1% for major osteoporotic fracture and 2.1% for hip fracture. She was taking monthly vitamin D supplementation. There was no documented history of antiresorptive therapy before or during the biochemical evaluation.

Laboratory evaluation demonstrated persistently elevated intact parathyroid hormone, reaching a peak of 101 pg/mL and remaining elevated on subsequent measurements (89.2 pg/mL and 71.1 pg/mL on two separate occasions), despite repeatedly normal serum calcium (preoperative value, 10.1 mg/dL). Serum phosphate was 4.2 mg/dL and 25-hydroxyvitamin D was within the sufficient range at 41.9 ng/mL. Renal function was normal (creatinine, 0.6–0.7 mg/dL). A 24-hour urine study showed hypercalciuria at 332 mg/24 h.

Neck ultrasound identified a well-circumscribed, hypoechoic, solid-appearing mass measuring approximately 0.7 × 0.6 × 0.5 cm inferior to the left thyroid lobe, raising suspicion of a parathyroid adenoma. The thyroid gland was otherwise unremarkable, with only small non-suspicious bilateral nodules. Technetium-99m sestamibi scintigraphy showed focal uptake at the inferoposterior margin of the left thyroid lobe, corresponding to the ultrasound finding and supporting a diagnosis of left inferior parathyroid adenoma.

In view of the biochemical findings, skeletal manifestations, and concordant imaging, the patient underwent left inferior parathyroidectomy with intraoperative frozen section evaluation. Gross assessment revealed a tan-pink nodular mass measuring approximately 1.6 × 1.0 × 0.5 cm, containing a cystic component measuring 1.3 cm filled with clear fluid. Frozen section showed parathyroid tissue with a unilocular cyst (Figure 1). Microscopic examination showed a cyst lined by flattened to cuboidal parathyroid cells, consistent with a parathyroid cyst, and no malignant features were identified (Figure 2).

**Figure 1:**
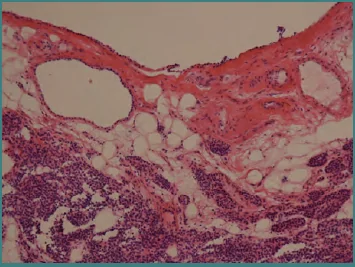
Frozen section of the excised left inferior parathyroid lesion showing parathyroid tissue with a unilocular cyst

**Figure 2:**
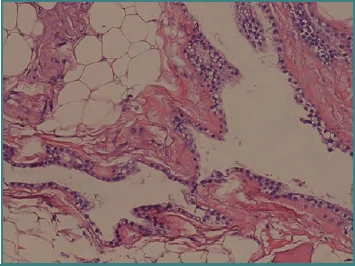
Higher-power view of the cyst showing a lining of flattened to cuboidal parathyroid cells, without malignant features

The patient remained clinically stable postoperatively. Parathyroid hormone decreased to 50.1 pg/mL after surgery, with a subsequent measurement of 57.5 pg/mL during follow-up. Total serum calcium remained within normal limits (9.4 mg/dL). She remained under endocrine follow-up for ongoing management of her skeletal disease and monitoring of parathyroid function.

## DISCUSSION

Parathyroid cysts are uncommon lesions and can be categorized as functional or non-functional according to their ability to secrete parathyroid hormone [1]. Functional parathyroid cystic lesions are exceedingly uncommon and are typically associated with elevated parathyroid hormone [1,2]. The pathogenesis is not fully understood; proposed hypotheses include cystic transformation of parathyroid adenomas, embryological remnants, and retention-type cysts [1,6].

Diagnosing normocalcemic primary hyperparathyroidism requires excluding any possible secondary cause of parathyroid hormone elevation. Antiresorptive therapy, for example, may provoke a compensatory rise in parathyroid hormone, usually accompanied by a transient decline in serum calcium; in the present case, no antiresorptive exposure was documented.

Establishing a definitive preoperative diagnosis of a parathyroid cyst can be difficult. Neck ultrasonography and technetium-99m sestamibi scintigraphy are commonly used to assess parathyroid disease; however, cystic parathyroid lesions may share imaging characteristics with parathyroid adenomas [2,6]. On ultrasound, parathyroid cystic lesions typically appear well-circumscribed and hypoechoic or anechoic, whereas sestamibi radiotracer uptake can be variable, particularly in functional cysts [2,3]. Consequently, imaging findings alone are usually insufficient to distinguish cystic lesions from adenomas [2,3]. Definitive diagnosis is usually confirmed by microscopic assessment after surgical excision [1,6].

Macroscopically, parathyroid cystic lesions may appear as thin-walled cystic structures containing clear or, less commonly, hemorrhagic fluid. Microscopically, they are lined by parathyroid tissue and may show cystic transformation without features of malignancy. Fine-needle aspiration with measurement of parathyroid hormone in the cyst fluid has been reported as a helpful diagnostic adjunct, particularly when preoperative differentiation from thyroid cystic lesions is unclear [6,7].

Management of parathyroid cysts depends on hormonal status and clinical presentation. Asymptomatic non-functional cysts may be managed conservatively, whereas complete surgical excision is generally recommended for functionally active cysts and for those causing compressive symptoms [1,7,8]. Surgery is both diagnostic and therapeutic in selected patients and is preferred for symptomatic or possibly functional lesions.

## CONCLUSION

Parathyroid cysts represent an uncommon and often diagnostically challenging form of parathyroid pathology. Their clinical, biochemical, and radiologic characteristics may closely resemble those of parathyroid adenomas, which makes preoperative differentiation difficult. Parathyroid cystic lesions may be associated with biochemical findings suggestive of hyperparathyroidism despite normal serum calcium and may be accompanied by clinically significant skeletal disease. These observations highlight the importance of considering parathyroid cysts in the differential diagnosis of parathyroid lesions, even when the biochemical findings are atypical. In the present case, microscopic examination established the diagnosis. Serum calcium remained within the normal range postoperatively, and parathyroid hormone showed only a mild reduction; therefore, continued endocrinology follow-up was recommended.

## Data Availability

The data supporting the findings of this case report are contained within the article.
